# Loss-of-function of an *Arabidopsis* NADPH pyrophosphohydrolase, AtNUDX19, impacts on the pyridine nucleotides status and confers photooxidative stress tolerance

**DOI:** 10.1038/srep37432

**Published:** 2016-11-22

**Authors:** Takanori Maruta, Takahisa Ogawa, Masaki Tsujimura, Keisuke Ikemoto, Tomofumi Yoshida, Hiro Takahashi, Kazuya Yoshimura, Shigeru Shigeoka

**Affiliations:** 1 Department of Advanced Bioscience, Faculty of Agriculture, Kindai University, 3327-204 Nakamachi, Nara 631-8505, Japan; 2 Department of Life Science and Biotechnology, Faculty of Life and Environmental Science, Shimane University, 1060 Nishikawatsu, Matsue, Shimane 690-8504, Japan; 3 Graduate School of Horticulture, Chiba University, 648 Matsudo, Matsudo, Chiba 271-8510, Japan; 4 Department of Food and Nutritional Science, College of Bioscience and Biotechnology, Chubu University, 1200 Matsumoto-cho, Kasugai, Aichi 487-8501, Japan (K.Y.)

## Abstract

The levels and redox states of pyridine nucleotides, such as NADP(H), regulate the cellular redox homeostasis, which is crucial for photooxidative stress response in plants. However, how they are controlled is poorly understood. An *Arabidopsis* Nudix hydrolase, AtNUDX19, was previously identified to have NADPH hydrolytic activity *in vitro*, suggesting this enzyme to be a regulator of the NADPH status. We herein examined the physiological role of AtNUDX19 using its loss-of-function mutants. NADPH levels were increased in *nudx19* mutants under both normal and high light conditions, while NADP^+^ and NAD^+^ levels were decreased. Despite the high redox states of NADP(H), *nudx19* mutants exhibited high tolerance to moderate light- or methylviologen-induced photooxidative stresses. This tolerance might be partially attributed to the activation of either or both photosynthesis and the antioxidant system. Furthermore, a microarray analysis suggested the role of ANUDX19 in regulation of the salicylic acid (SA) response in a negative manner. Indeed, *nudx19* mutants accumulated SA and showed high sensitivity to the hormone. Our findings demonstrate that ANUDX19 acts as an NADPH pyrophosphohydrolase to modulate cellular levels and redox states of pyridine nucleotides and fine-tunes photooxidative stress response through the regulation of photosynthesis, antioxidant system, and possibly hormonal signaling.

The nicotinamide pyridine nucleotides, NAD(H) and its derivative NADP(H), play pivotal roles as redox compounds; they are ubiquitous co-factors that are required for hundreds of redox reactions in a number of metabolic pathways, including photosynthesis and the antioxidant system^1^. Pyridine nucleotides as well as reactive oxygen species (ROS) and antioxidants are therefore crucial determinants of cellular and organellar redox homeostasis, which modulate stress acclimation as well as growth and development in plants^1^,^2^,^3^.

In plant chloroplasts, the levels and redox states of NADP(H) are the determinant of ROS production by the photosynthetic transport chain (PET). The depletion of NADP^+^ as an electron acceptor, which often occurs under high light irradiation, excessively reduce PET, resulting in the facilitation of oxygen reduction followed by ROS production (photooxidative stress)^4^,^5^. Plants have evolved a number of mechanisms, including antioxidant systems and light energy dissipation as heat (xanthophyll cycle), thereby avoiding or acclimating to the oxidative stress caused by illumination^4^,^5^,^6^,^7^. NADPH also serves as the source of reducing equivalents for the ascorbate-glutathione cycle and the thioredoxin-dependent network, two major antioxidant systems in plant cells^2^, and also for the xanthophyll cycle, because this requires ascorbate, which is recycled using ferredoxin or NADPH. Thus, the redox states of NADP(H) are crucial for regulating both ROS production and scavenging in chloroplasts.

Pyridine nucleotides have also been characterized as signals that regulate stress and hormonal responses in plants. In *Arabidopsis* leaves, treatments with NAD^+^, NADH, NADP^+^, and NADPH enhanced the levels of salicylic acid (SA), a phytohormone essential for the biotic stress response, and the expression of *PATHOGENESIS-RELATED1* (*PR1*)^8^, a representative SA-responsive gene. Since the plasma membrane is highly impermeable to pyridine nucleotides^1^, these findings indicate that extracellular pyridine nucleotides act as signals to induce the SA-dependent biotic stress response. Pétriacq *et al*.^9^ recently created transgenic *Arabidopsis* plants that overexpressed a bacterial *quinolinate phosphoribosyltransferase* gene. In the transgenic plants, a quinolinate treatment increased the intracellular levels of all pyridine nucleotides, especially NAD^+^, resulting in enhanced SA levels and *PR1* expression. Thus, both intracellular and extracellular pyridine nucleotides positively regulate the SA pathway. Pyridine nucleotides also serve as the precursors for cyclic ADP-ribose and nicotinic acid adenine dinucleotide phosphate, intracellular Ca^2+^-mobilizing agents, which promotes the release of Ca^2+^ from stores^10^. In addition to their function in the cellular redox homeostasis, these facts indicate that the levels and redox states of pyridine nucleotides must be tightly controlled to fine-tune plant responses to stress and hormone.

A number of metabolic pathways, including primary metabolisms and synthesis of pyridine nucleotides, are involved in the regulation of pyridine nucleotides levels and redox states. The pyridine nucleotide synthesis and its role in photooxidative stress response have been addressed. For example, *Arabidopsis* NAD kinase 2 (AtNADK2), which catalyzes the phosphorylation of NAD^+^ to produce NADP^+^ in chloroplasts, has been shown to be essential for normal growth, photosynthesis, and photooxidative stress tolerance^11^,^12^. However, the mode of action of pyridine nucleotide catabolism and its physiological role are poorly understood. One of candidates involved in the catabolic process is Nudix (nucleoside diphosphates linked to some moiety X) hydrolase family, having hydrolytic activity toward various nucleoside diphosphate derivatives, which include NADH and NADPH, and being widely distributed among all classes of organisms^13^,^14^,^15^,^16^. *Arabidopsis* possesses 28 Nudix hydrolases (AtNUDXs) and some isoforms that have NADH hydrolase activity, such as AtNUDX6 and AtNUDX7, though the latter also uses ADP-ribose as an alternative substrate^17^. AtNUDX6 and 7 are known to act as positive and negative regulators, respectively, of SA-induced gene expression^18^,^19^,^20^,^21^,^22^,^23^. This provides further evidence that the regulation of cellular NADH levels through Nudix enzymes is critical for SA-mediated responses.

Among the different AtNUDXs, AtNUDX19 (At5g20070), which is known to be targeted to both chloroplasts and peroxisomes^24^,^25^, is currently the only enzyme to exhibit pyrophosphohydrolase activity toward NADPH *in vitro*^24^, suggesting that this enzyme participates in plant response to photooxidative stress by regulating the NADPH levels. Indeed, AtNUDX19 was very recently found to regulate NADPH levels and activity of enzymes involved in the NADPH production in *Arabidopsis* leaves and roots^26^. We herein examined the physiological roles of AtNUDX19 and found that this enzyme is a new regulator of the levels and redox states of pyridine nucleotides and fine-tunes the photooxidative stress response in plant cells.

## Results

### Structure and distribution of AtNUDX19 type enzymes in plants

**Figure 1:**
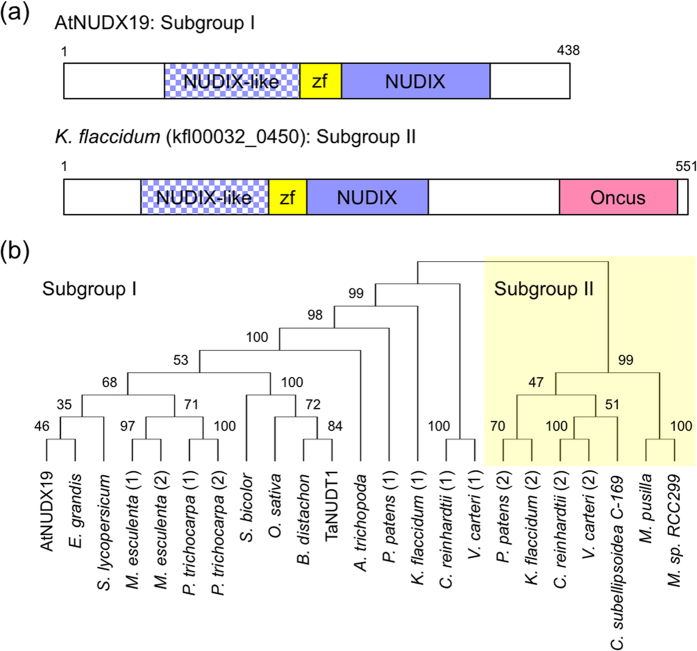
Structure and phylogenetic tree of AtNUDX19-type (SQPWP) enzymes. (**a**) Structure of subgroup I (AtNUDX19) and II (*Klebsormidium flaccidum*, kfl00032_0450) enzymes. (**b**) SQPWP sequences from various photosynthetic eukaryotes, which are *Arabidopsis thaliana* (AtNUDX19), *Triticum aestivum L*. (TaNUDT1), *Eucalyptus grandis*, *Manihot esculenta*, *Populus trichocarpa*, *Solanum lycopersicum*, *Amborella trichopoda*, *Sorghum bicolor*, *Brachypodium distachyon*, *Oryza sativa*, *Physcomitrella patens*, *Klebsormidium flaccidum*, *Volvox carteri*, *Chlamydomonas reinhardtii*, *Coccomyxa subellipsoidea C-169*, *Micromonas pusilla CCMP1545*, and *Micromonas sp. RCC299*. Accession numbers for these sequences are available in Supplemental Table S1. The phylogenetic tree was generated by the neighbor-joining method with 1,000 bootstraps using MEGA7 software.

Compared to other Nudix isoforms, AtNUDX19 and its homologues have been poorly characterized in plants. Therefore, we started with analyses of structure, distribution, and evolutional history of this new enzyme. Using the Pfam database (version 29.0)^27^, we found that AtNUDX19 consists of three domains; i.e., NADH pyrophosphatase-like rudimentary NUDIX domain (NUDIX-like, PF09296), NADH pyrophosphatase zinc ribbon domain (zf-NADH-PPase: PF09297), and NUDIX domain (PF00293) (Fig. 1a). An obvious Nudix motif (GX_5_EX_7_REUXEEXGU)^16^ was observed in the NUDIX domain (Supplemental Figure S1), but not in the NUDIX-like, probably suggesting the later domain to have no hydrolase activity. The zf-NADH-PPase domain was located between the NUDIX-like and NUDIX domains. AtNUDX19 is known to have the SQPWPFPxS motif 28 immediately downstream of the Nudix motif within the NUDIX domain (see Supplemental Figure S1). According to previous classification of *Arabidopsis* isoforms^29^, only AtNUDX19 belongs to the NADH pyrophosphohydrolase group. However, the group name ‘NADH pyrophosphohydrolase’ is inadequate to explain the function of AtNUDX19 owing to the following reasons; (1) other isoforms in the FGFTNE (fibroblast growth factor) group, such as AtNUDX6 and 7, also have NADH pyrophosphohydrolase activity^17^, and (2) AtNUDX19 has little activity toward NADH *in vivo* (see the following results and discussion). Therefore, we refer this as the ‘SQPWP’ group in this study because of the presence of the SQPWPFPxS motif, which is lacked in isoforms of other groups^29^.

We then examined the sequenced genomes of various plant species, including algae, moss, and higher plants, for the presence of the SQPWP enzyme(s). All photosynthetic eukaryotes analyzed possessed more than one isoform (Supplemental Table S1), while no photosynthetic prokaryotes with this group was found (using Cyanobase)^30^; only one exception was *Rhodopseudomonas palustris CGA009*, photosynthetic bacteria, which had the SQPWP enzyme (RPA0613, data not shown). Like AtNUDX19, these enzymes had the NUDIX, NUDIX-like, and zf-NADH-PPase domains and the Nudix and SQPWPFPxS motifs with some exceptions (see Supplemental Table S1). Thus, the SQPWP group is widely distributed in photosynthetic eukaryotes, although it remains to be experimentally addressed whether these can use pyridine nucleotides as substrates. Phylogenetic analysis revealed that their sequences could be divided into further 2 subgroups (I and II) (Fig. 1b). Higher plants contained only subgroup I, while moss (*Physcomitrella patens*), the charophyte (*Klebsormidium flaccidum*), and the chlorophytes (*Chlamydomonas reinhardtii* and *Volvox carteri*) had both subgroups. In contrast, the trebouxiophyte (*Coccomyxa subellipsoidea C-169*, also known as *Chlorella vulgaris*) and the prasinophytes (*Micromonas pusilla CCMP1545* and *Micromonas sp. RCC299*) possessed only subgroup II. These findings suggest that subgroup II was ancestral and then lost in higher plants, which have another subgroup I acquired in the chlorophytes. All enzymes in subgroup II, except for the *Micromonas* enzymes, had an additional Oncus domain (Supplemental Table S1), which was found in the testes-specific Janus/Ocnus family proteins in *Drosophila melanogaster*^31^. Almost subgroup I enzymes possessed both chloroplast- and peroxisome-targeting signals (Supplemental Table S2). Indeed, AtNUDX19 was experimentally confirmed to be localized in both organelles^24^,^25^.

### The levels and redox states of Pyridine nucleotides in *nudx19* mutants

To investigate the physiological function of AtNUDX19, *Arabidopsis* lines (SALK_115339 and SALK_135053) containing a T-DNA insert in the *AtNUDX19* gene were used in this study. Both lines contained a T-DNA in the fourth intron region of the gene (Supplemental Figure S2). As described in Corpas *et al*.^26^, neither transcript nor protein of AtNUDX19 was detected in the homozygous SALK_135053 line (Supplemental Figure S2). In contrast, very low levels of the AtNUDX19 transcript and protein were observed in the homozygous SALK_115339 line. Thus, the SALK_135053 and SALK_115339 lines were knockout (KO-*nudx19*) and knockdown (KD-*nudx19*) mutants of this gene, respectively. The growth of these mutants was similar to that of wild-type plants under normal growth conditions (16 h of 100 μmol photons m^−2^ s^−1^, 8 h of dark).

**Figure 2:**
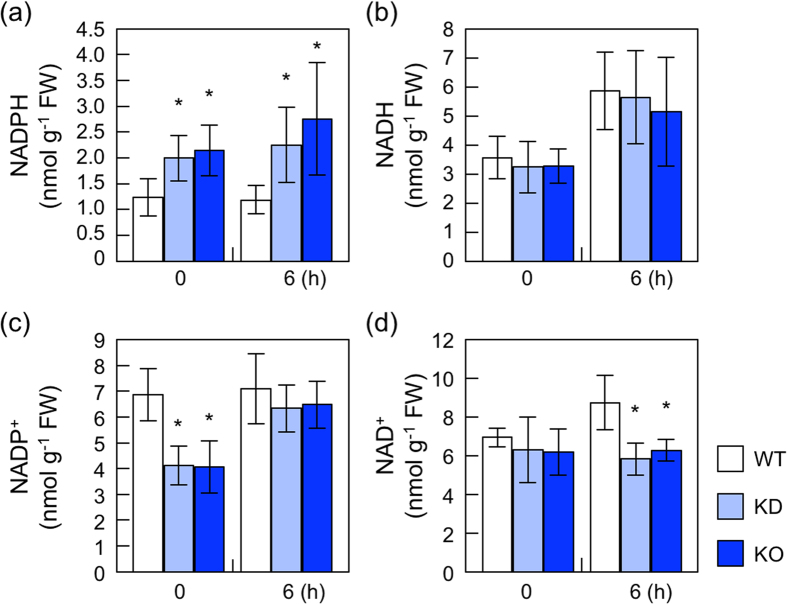
Pyridine nucleotides levels in wild-type and *nudx19* mutants. Three-week-old wild-type (WT) and mutant plants (KD and KO) were exposed to high light (1,000 μmol photons m^−2^ s^−1^) for 6 h. The levels of pyridine nucleotides, NADPH (**a**), NADH (**b**), NADP^+^ (**c**), and NAD^+^ (**d**), in leaves were measured. Data are means ± SD for at least 3 individual experiments (≥5 plants for 1 experiment). Significant differences: *P < 0.05 vs. the value of wild-type plants.

We then investigated the effects of disrupting AtNUDX19 on pyridine nucleotides levels in leaves. Three-week-old wild type and *nudx19* mutants grown under normal light conditions were exposed to high light (1,200 μmol photons m^−2^ s^−1^) for 6 h. Under both normal and high light intensities, NADPH levels were significantly higher in both KD- and KO-*nudx19* plants than in the wild-type plants (Fig. 2). Interestingly, NADP^+^ levels were lower in KD- and KO-*nudx19* plants under normal light, but recovered to the wild-type levels by high light exposure. As a consequent, total NADP(H) levels in the *nudx19* mutants were lower than those in the wild-type plants under normal light, but slightly higher after high light exposure (Table 1). The ratio of NADPH to total NADP(H) in KD- and KO-*nudx19* plants was approximately 2-fold higher than that in wild type under both conditions. The lack of AtNUDX19 had no impact on NADH levels (Fig. 2). Under normal light intensity, there was also no difference in NAD^+^ levels between wild-type and mutant plants. Although NAD^+^ levels were increased in response to high light in wild type, this increase was almost completely inhibited in the *nudx19* mutants (Fig. 2), which increased the ratio of NADH to total NAD(H) (Table 1). Together with its enzymological properties obtained from *in vitro* assay^24^, current findings demonstrate that AtNUDX19 catalyzes the hydrolysis of NADPH, but not NADH, *in vivo* and has significant impacts on the cellular levels and redox states of pyridine nucleotides.

We also checked the effect of light intensity on the AtNUDX19 expression. When 1-week-old wild-type plants were further grown under different light intensities (16 h of 20, 100, and 800 μmol photons m^−2^ s^−1^, 8 h of dark) for 2 weeks, the increase in the intensity of growth light had positive effect on the transcript levels of *AtNUDX19* but not on its protein levels (Supplemental Figure S3).

### Photooxidative stress tolerance of *nudx19* mutants

**Figure 3:**
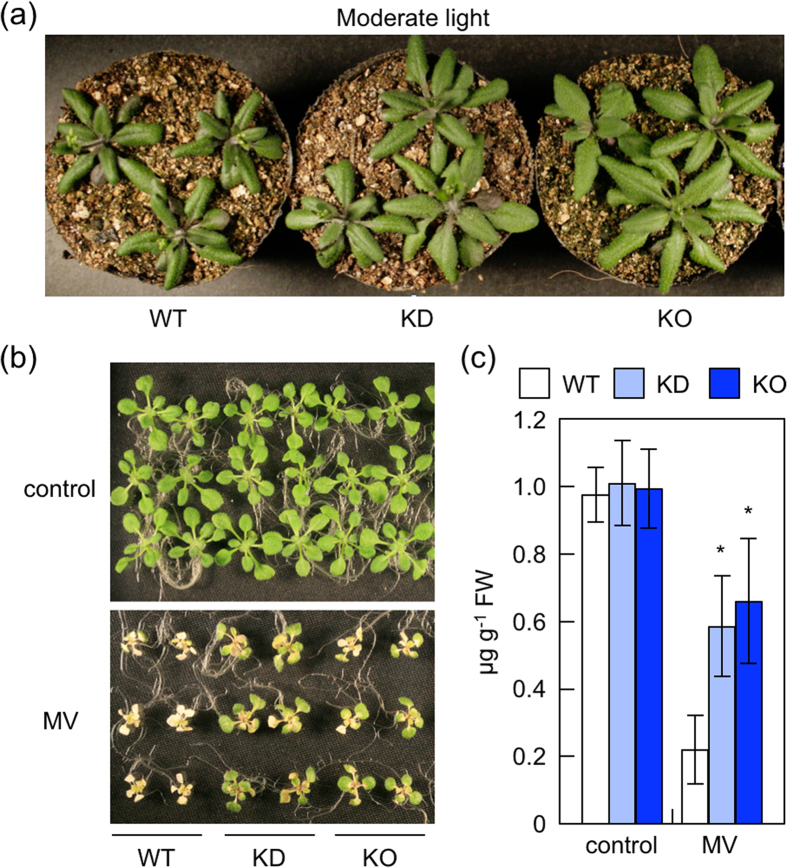
Tolerance of *nudx19* mutants to photooxidative stress. (**a**) One-week-old wild-type and mutants, grown on MS medium under normal light conditions, were transferred to soil and grown further under moderate light stress conditions (16 h of 400 μmol photons m^−2^ s^−1^, and 8 h of dark). Photograph of seedlings 2 weeks after transferring to moderate light conditions. The same results were obtained in 4 independent experiments. The results of the representative leaves were photographed. (**b,c**) Ten-day-old wild-type and mutants, grown on MS medium under normal light conditions, were transferred to medium containing 4 μM methylviologen (MV), an ROS generator, and grown further for 1 week under the same light conditions. (**b**) Photograph of seedlings 1 week after the MV treatment. The same results were obtained in 4 independent experiments. The results of representative leaves were photographed. (**c**) Chlorophyll contents in shoots. Data are means ± SD for 4 individual experiments (≥10 plants for 1 experiment). Significant differences: *P < 0.05 vs. the value of wild-type plants.

Before and after high light exposure, *nudx19* mutants showed high reduction state of NADPH (Table 1), which, in general, can excessive reduce PET, thereby enhancing ROS production^4^. Although we did not address whether the redox change occurred within chloroplasts, our findings implied that *nudx19* might be highly sensitive to photooxidative stress. However, when 1-week-old wild-type and *nudx19* plants were grown further under moderate light conditions (16 h of 450 μmol photons m^−2^ s^−1^, 8 h of dark) for 2 weeks, the growth of KO- and KD-*nudx19* plants was unexpectedly but clearly enhanced (Fig. 3a). Furthermore, KO- and KD-*nudx19* plants were highly tolerant to the treatment with methylviologen (MV), a ROS-producing agent in chloroplasts and mitochondria (Fig. 3b,c), suggesting the *nudx19* mutants to be insensitive to oxidative stress.

### Photosynthesis in *nudx19* mutants under high light

**Figure 4:**
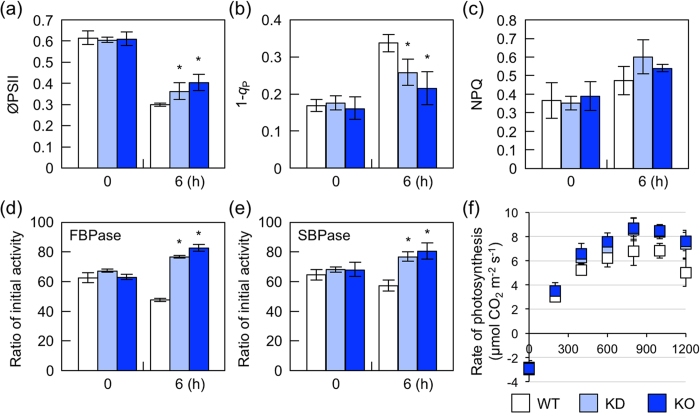
Photosynthetic parameters in *nudx19* mutants under high light. Three-week-old wild-type and mutant plants were exposed to high light (1,000 μmol photons m^−2^ s^−1^). (**a–c**) øPSII, 1-*q*_P_, and NPQ in leaves was determined at 25 °C after dark adaptation for 15 min. Data are means ± SD for 4 individual experiments. (**d, e**) The initial activities of FBPase and SBPase in leaves were measured. Data are means ± SD for 4 individual experiments (≥3 plants for 1 experiment). Significant differences: *P < 0.05 vs. the value of wild-type plants. (f) CO_2_ fixation was measured in leaves with an LI-6400 portable photosynthesis system. Each data point represents the mean ± SD of at least 5 individual experiments.

We hypothesized that the tolerance of *nudx19* mutants to photooxidative stress was due to an enhancement in photosynthesis, increase in antioxidant capacity, and/or modulation of the expression of defense genes through changes in the levels and redox states of NAD(P)(H). To clarify the effects of disrupting AtNUDX19 on PET, the quantum yield of photosystem II (øPSII) and photochemical and nonphotochemical quenching (*q*_P_ and NPQ, respectively) were measured under high light in wild-type and *nudx19* plants (Fig. 4). 1-*q*_P_ indicates the reduction states of PSII^32^. In all genotypes, 1-*q*_P_ and NPQ were increased by high light irradiation (6 h), whereas øPSII was decreased. However, the increase in 1-*q*_P_ was mitigated in KO- and KD-*nudx19* plants, although the lack of AtNUDX19 had no significant effect on NPQ (Fig. 4). The decrease in øPSII was also suppressed in KO- and KD-*nudx19* plants (Fig. 4). These results indicate that *nudx19* mutants could prevent the excessive reduction of PET more than wild-type plants.

We also examined the activities of enzymes involved in the Calvin cycle, sedoheptulose-1,7-bisphosphatase (SBPase) and fructose-1,6-bisphosphatase (FBPase), which are tightly controlled through thiol-dependent redox regulation. Although the lack of AtNUDX19 had no effect on the total activities of SBPase and FBPase under normal and high light conditions (data not shown), the initial activities of these enzymes were significantly higher in *nudx19* mutants than in wild-type plants during high light exposure (Fig. 4). In line with this, the carbon assimilation rate was also higher in *nudx19* mutants than in wild-type plants above a light intensity of 200 μmol photons m^−2^ s^−1^, especially at a saturating irradiance (1,200 μmol photons m^−2^ s^−1^).

### Activity of antioxidant enzymes in *nudx19* mutants

**Figure 5:**
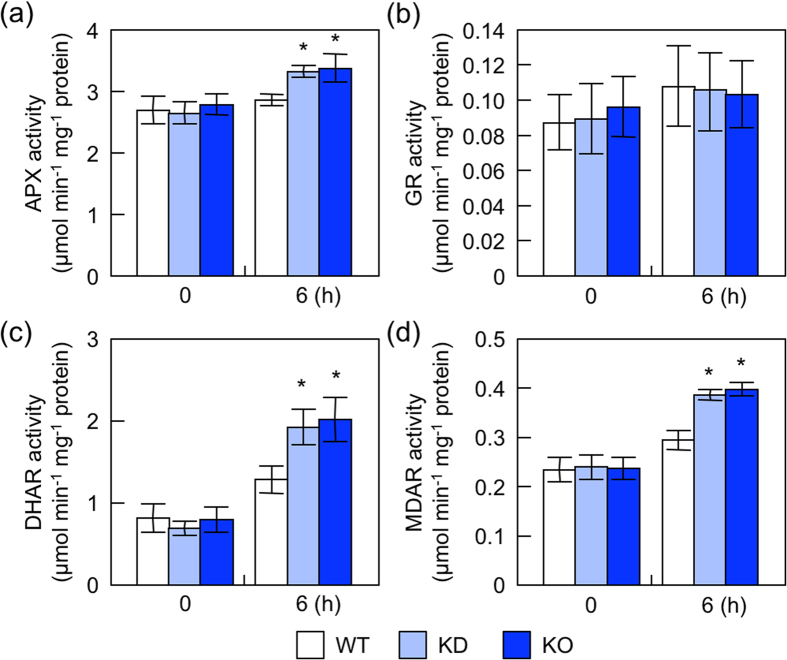
Activities of antioxidative enzymes in *nudx19* mutants under high light. Three-week-old wild-type and mutant plants were exposed to high light (1,000 μmol photons m^−2^ s^−1^) for 6 h. The activities of APX (**a**), GR (**b**), DHAR (**c**), and MDAR (**d**) in leaves were measured. Data are means ±SD for 4 individual experiments (≥3 plants for 1 experiment). Significant differences: *P < 0.05 vs. the value of wild-type plants.

We then measured the total activities of antioxidative enzymes, ascorbate peroxidase (APX), dehydroascorbate reductase (DHAR), monodehydroascorbate reductase (MDAR), and glutathione reductase (GR), all of which are components of the ascorbate-glutathione cycle, one of the main systems for scavenging ROS in plant cells^2^,^3^,^4^. Before high light exposure, no significant difference was observed in the activities of any of these enzymes between wild-type and *nudx19* plants (Fig. 5). However, all the activities, except for GR, were slightly but significantly higher in KO- and KD-*nudx19* plants than in wild-type plants after 6 h of high light exposure (Fig. 5). These results suggest that the disruption of AtNUDX19 activates both photosynthetic and antioxidative capacities under high light, possibly leading to higher tolerance to the stress.

### Effects of AtNUDX19-disruption on nuclear gene expression

A microarray analysis was performed using wild-type and KO-*nudx19* leaves under normal growth conditions to investigate the effects of the altered NADPH status on gene expression. Differences in the change ratio of gene expression were evaluated by the Wilcoxon signed-rank test and multiple testing problems were corrected by the Benjamini-Hochberg method^33^. Genes with expression levels that were 1.5-fold higher (37 genes) or lower (16 genes) in KO-*nudx19* leaves than in wild-type leaves were selected (Supplemental Table S2). No gene encoding antioxidative enzyme, such as *APX*, *DHAR*, *MDAR*, and *GR*, was included in the up-regulated genes. Furthermore, only one gene among the up-regulated genes, light harvesting complex 1 (*LHCA1*), encoded a photosynthesis-related gene.

**Figure 6:**
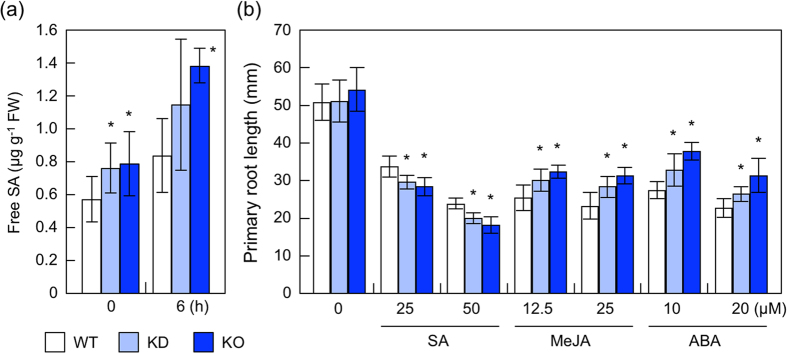
Effect of *nudx19* mutations on SA levels and sensitivity to hormones. (**a**) Three-week-old wild-type and mutant plants were exposed to high light (1,000 μmol photons m^−2^ s^−1^) for 6 h. Free SA levels in leaves were measured. Data are means ± SD for 6 individual experiments (≥5 plants for 1 experiment). (**b**) Three-day-old wild-type and mutant plants, grown on MS medium, were transferred to and further grown on medium containing SA (25 and 50 μM), MeJA (12.5 and 25 μM), and ABA (10 and 20 μM). Root length was measured 5 days after the treatment. Data are means ± SD for at least 3 individual experiments (≥10 seedlings for 1 experiment). Significant differences: *P < 0.05 vs. the value of wild-type plants.

We found 2 representative SA-responsive genes, *PR1* and *PR2*, among the up-regulated genes (Supplemental Table S2). In addition, the transcript levels of *SYSTEMYC AQUIRED RESISTANCE DEDICIENT1* (*SARD1*) and the *WRKY38* transcription factors, which are known to be involved in the SA-dependent pathogen response^20^,^34^,^35^, were enhanced in KO-*nudx19* leaves (Supplemental Table S2). Notably, SARD1 binds to the promoter of *SALICYLIC ACID INDUCTION DEFICIE2* (*SID2*) gene encoding an isochorismate synthase, which is required for SA biosynthesis in *Arabidopsis*, and activates its expression^35^. Therefore, we investigated the effects of *nudx19* mutations on the levels of and sensitivity to SA. The levels of free SA were significantly enhanced in *nudx19* mutants under normal and high light (Fig. 6a). Furthermore, when 3-day-old plants were grown on MS medium containing SA (25 and 50 μM) for a further 5 days, KO- and KD-*nudx19* plants were slightly but significantly more sensitive to the treatment than wild-type plants (Fig. 6b). We also found that the expression of AtNUDX19 was responsive to the SA treatment (Supplemental Figure S4). These results indicate that AtNUDX19 acts as a negative regulator of SA synthesis. In contrast, KO- and KD-*nudx19* plants were slightly insensitive to jasmonic acid methyl (MeJA) and abscisic acid (ABA) (Fig. 6b). This might be explained by the fact that SA acts as an antagonist of these hormones^36^,^37^. The expression of AtNUDX19 was also responsive to treatments with these hormones (Supplemental Figure S4).

## Discussion

Here, we addressed the physiological function of AtNUDX19, which is widely distributed throughout photosynthetic eukaryotes (Fig. 1), in photooxidative stress response using its loss-of-function mutants. Our findings indicate that 1) only NADPH is a physiological substrate of the enzyme *in vivo*, (2) AtNUDX19 regulates cellular levels and redox states of pyridine nucleotides, and (3) this regulation is associated with plant responses to photooxidative stress and hormones.

Our previous *in vitro* assay showed that a recombinant AtNUDX19 protein can hydrolyze both NADPH and NADH, but its *K*_m_ value for NADPH (36.9 μM) is approximately 9-fold lower than that for NADH (335.3 μM), suggesting that AtNUDX19 prefers NADPH to NADH as a substrate^24^. Similarly, a wheat Nudix hydrolase (TaNUDT1) strongly prefers NADPH^38^. In line with these findings, the present study showed that the levels of NADPH, but not other pyridine nucleotides, were enhanced in AtNUDX19-disrupted mutants (Fig. 2). These results strongly indicate AtNUDX19 to hydrolyze NADPH, but not NADH, *in vivo*. As described above, AtNUDX19 is targeted to both chloroplasts and peroxisomes^24^,^25^. In chloroplast stroma, the concentration of NADPH has been estimated to be 0.29 to 0.5 mM in light and 0.12 mM in darkness^39^,^40^, whereas that in peroxisomes is presently unknown. Furthermore, 60–85% of total cellular NADPH is present in illuminated chloroplasts^41^. Thus, the chloroplastic localization of AtNUDX19 is likely to be suitable for its enzymatic reaction.

The lack of AtNUDX19 had significant impacts on the levels and redox states of pyridine nucleotides (Fig. 2). For example, levels of NADP^+^ were lower in the mutants than in wild type, resulting in a decrease in total NADP(H) under normal light. It is currently difficult to provide an exact explanation how AtNUDX19 affects the pyridine nucleotides status in plant cells, because their redox states are finely tuned through various metabolic pathways. One of possible explanations might be provided by a recent finding that the lack of AtNUDX19 stimulated the activity of enzymes involved in the NADPH production from NADP^+^; i.e., NADP-isocitrate dehydrogenase, NADP-malicenzyme, glucose-6-phosphate dehydrogenase and 6-phosphogluconate dehydrogenase^26^. These enzymes might be activated to produce more NADPH in *nudx19* mutants, which resulted in the decrease of NADP^+^.

One of unexpected results was that despite a high NADPH/NADP^+^ ratio, *nudx19* mutants were highly tolerant to photooxidative stresses (Figs 2 and 3 and Table 1). The measurement of chlorophyll fluorescence indicated that the *nudx19* mutations could prevent the excessive reduction of PET (Fig. 4). This might be partially due to the activation of photosynthetic carbon assimilation and antioxidant systems in the *nudx19* mutants (Figs 4 and 5), because these pathways can act as electron sink for PET^4^. Although how these systems were activated in *nudx19* mutants is currently unknown, NADPH is known to function as a positive effector to promote the activation of ribulose 1,5-bisphosphate carboxylase/oxygenase (Rubisco), a key enzyme of the Calvin cycle^42^,^43^. The activation of photosynthesis was also observed in transgenic *Arabidopsis* and rice lines overexpressing chloroplastic NADK2^44^,^45^. Unlike NADK2-overexpressing plants, in which the levels of both NADP^+^ and NADPH were increased^44^,^45^, the *nudx19* mutants only accumulated NADPH and no other pyridine nucleotides (Fig. 2). Assuming that photosynthesis is enhanced through the same mechanism in both *nudx19* mutants and NADK2-overexpressors, these findings suggest that NADPH is more critical for the enhancement of photosynthesis than NADP^+^.

A microarray analysis revealed that expression of approximately 50 genes was affected in *nudx19* mutants (Supplemental Table S2), allowing us to expect that these changes might also be associated with the photooxidative stress tolerance of the mutants. Interestingly, two of up-regulated genes were *PR1* and *PR2*, which was in line with the elevated levels of SA and high sensitivity to the hormone (Fig. 6). As described above, intracellular and extracellular pyridine nucleotides can activate the SA pathway irrespective of the kinds of pyridine nucleotides^8^,^9^. These observations indicate that the enhanced NADPH levels in these mutants activate SA biosynthesis, and that AtNUDX19 acts as a negative regulator of SA biosynthesis and its response. Since controlled levels of SA were also previously shown to be required for optimal photosynthesis^46^, the enhanced SA pathway might affect the abilities of photosynthesis and antioxidant defense in *nudx19* mutants. In agreement with previous findings that SA is an antagonist of JA and ABA^36^,^37^, *nudx19* mutants showed an insensitivity to MeJA and ABA (Fig. 6), although it should be experimentally investigated if the insensitivity was due to the activation of the SA pathway.

Taken together, our results demonstrate that AtNUDX19 is a novel regulator of the pyridine nucleotides status to modulate plant response to photooxidative stress and hormones, although more studies are required to elucidate the mechanism(s) underlying how the pyridine nucleotides status modulate such responses. Since other Nudix hydrolases having NADH (but not NADPH) hydrolytic activity, such as AtNUDX6 and 7, are also involved in the photooxidative response in a positive manner, it will also be interesting to investigate the crosstalk between these AtNUDX isoforms and AtNUDX19 in the fine-tuning of stress and hormonal responses in future studies.

## Methods

### Plant materials and growth conditions

*Arabidopsis thaliana* (Col-0) was used as wild type in this study. Two T-DNA insertion lines for *AtNUDX19* gene (SALK_135053 and SALK_115339) were obtained from Arabidopsis Biological Resource Centre. To achieve normal growth, surface-sterilized seeds were sown on Murashige and Skoog (MS) medium containing 3% sucrose. Plates were stratified in darkness for 2 or 3 days at 4 °C and then transferred to a growth chamber kept at 23 °C during 16 h of light (100 μmol photons m^−2^ s^−1^) and at 22 °C during 8 h of darkness. After 7 days, seedlings were potted in soil and grown under the same conditions. The methods used for the applications of stresses and hormones are described in the figure legends.

### Preparation of total RNA, cDNA synthesis, and semi-quantitative RT-PCR analysis

Total RNA was isolated from the leaves of *Arabidopsis* plants using Sepasol-RNA I (Nacalai Tesque, Kyoto, Japan). First strand cDNA was synthesized using reverse transcriptase (ReverTra Ace; Toyobo) with an oligo dT primer. These analyses were performed according to the manufacturer’s instructions. A semi-quantitative RT-PCR analysis was performed according to Ogawa *et al*.^17^. Primer sequences are as follows; AtNUDX19-F (5′-ATGCTTGCTCTCTTCCTCTC-3′), AtNUDX19-R (5′-CTACAAACTTGAGAGAGAGACACC-3′), Actin8-F (5′-GAGATCCACATCTGCTGG-3′), and Actin8-R (5′-GCTGAGAGATTCAGGTGCCC-3′).

### Quantitative RT-PCR (q-PCR) analysis

Primer pairs for q-PCR were designed using PRIMER EXPRESS software (Applied Biosystems). Primer sequences are as follows; AtNUDX19-QF (5′-TTTGGCAGAGGATGGTTTCG-3′), AtNUDX19-QR (5′-GCCAACTCATCCATAGCACGTT-3′), Actin2-QF (5′-GGTGGTTCCATTCTTGCTTCCC-3′), Actin2-QR (5′-TCATACTCGGCCTTGGAGATCC-3′). Gene-specific primers were chosen such that the resulting PCR product had an approximately equal size of 100 bp. q-PCR was performed with an Applied Biosystems 7300 Real Time PCR System, using the SYBR Premix Ex Taq (Takara). *Actin2* mRNA was used as an internal standard in all experiments.

### Western blotting

The Western blot analysis was carried out as described previously^24^. The protein bands were detected using a polyclonal rabbit antibody (anti-AtNUDX19) prepared using the recombinant protein as the primary antibody and anti-rabbit IgG-HRP conjugate (Bio-Rad, CA) as the secondary antibody.

### Measurements of pyridine nucleotides and free SA

NAD^+^, NADH, NADP^+^, and NADPH levels were measured according to Ishikawa *et al*.^20^. Free SA levels were measured according to Maruta *et al*.^47^.

### Measurements of chlorophyll fluorescence and carbon assimilation rates

Chlorophyll fluorescence in *Arabidopsis* leaves was measured after dark adaptation for 20 min at 23 °C with a Junior-PAM (Waltz, Efeltrich, Germany). The actinic irradiance used was 120 (for normal light) or 400 μmol photons m^−2^ s^−1^ (for high light). The quenching parameters, *q*_P_ and NPQ, were calculated according to van Kooten & Snel^48^.

### Enzyme assays

The soluble fraction extracted from 0.2 g of *Arabidopsis* leaves was used for all enzyme assays. APX activity was measured as a decrease in absorbance at 290 nm (ε = 2.8 mM cm^−1^) due to the oxidation of ascorbate. Leaves were homogenized with 400 μl of 50 mM potassium phosphate (pH 7.5) containing 1 mM EDTA and 10% D-solbitol for the DHAR and GR assays, while leaves were homogenized with 400 μl of 50 mM potassium phosphate (pH 7.5) containing 0.2 mM EDTA, 10 mM 2-mercaptoethanol, and 1% D-solbitol for the MDAR assay. After centrifugation (20,000 × g) for 20 min at 4 °C, the supernatant obtained was used for these enzyme assays. In the DHAR assay, 20 μl of extract was added to 1 ml of reaction mixture containing 50 mM potassium phosphate (pH 7.5), 2 mM GSH, and 1 mM DHA, 0.2 mM NADPH, and GR. glutathione-dependent dehydroascorbate reduction was monitored at 340 nm. In the MDAR assay, 20 μl of extract was added to 1 ml of reaction mixture containing 100 mM potassium phosphate (pH 7.5), 1 mM ascorbate, and 0.2 mM NADPH. The reaction was started by the addition of 0.2 unit ascorbate oxidase after pre-incubation for 2 min. NADPH-dependent MDAR activity was monitored at 340 nm. In the GR assay, 10 μl of extract was added to 1 ml of reaction mixture containing 100 mM Tris-HCl (pH 7.8), 0.5 mM oxidized glutathione, 0.05 mM NADPH, and 0.5 mM EDTA. The reaction was started by the addition of NADPH and GSSG-dependent NADPH oxidation was monitored at 340 nm.

To measure the initial activities of SBPase and FBPase, leaf tissues were homogenized with 400 μl of 100 mM Tris–HCl buffer (pH 8.0), 16 mM MgCl_2_, 1 mM EDTA, 2% (w/v) PVP, and 0.05% Triton X-100. The maximum activities of both enzymes were determined by preincubating crude extracts with 20 mM dithiothreitol (DTT). The activities of both enzymes were measured according to Tamoi *et al*.^49^.

### Microarray and data analyses

The quality and purity of RNA were confirmed for the microarray analysis with an Ultrospec 2100 pro (GE Healthcare UK Ltd). Total RNA samples were reverse-transcribed, yielding double-strand cDNA, which was transcribed *in vitro* in the presence of biotin-labeled nucleotides with an IVT Labeling Kit (Affymetrix Inc.), and purified. Labeled cRNA was fragmented and hybridized to Affymetrix ATH1 GeneChip arrays for 16 h at 45 °C according to Affymetrix protocols. Arrays were washed on an Affymetrix Fluidics Station 450 and measured for fluorescence intensity with an Affymetrix GeneChip Scanner 3000. Raw data were processed using Affymetrix Gene Chip Operating Software (GCOS; Version 1.4.0.036). Two biological replicates were analyzed. We initially calculated the log2 (ratio) of gene expression and *P*-values based on the one-sided Wilcoxon signed-rank test for *nudx19* mutants against Col-0 by GCOS. *Q* values (adjusted *P*-values) were calculated from *P*-values by the Benjamini-Hochberg method^33^. In this experiment, we excluded 64 controls and 2,191 genes subject to cross-hybridization, according to NetAffx Annotation (www.affymetrix.com). We finally selected 37 up-regulated genes and 16 down-regulated genes, these genes showed >1.5-fold and *q* < 0.20 commonly for both replication sets. The criteria of *q* < 0.20 is often used for corrections of multiple testing problems^50^.

## Additional Information

****Accession codes:**** The microarray data were deposited in the public NCBI Gene Expression Omnibus database under the GEO accession number GSE64968.

**How to cite this article**: Maruta, T. *et al*. Loss-of-function of an *Arabidopsis* NADPH pyrophosphohydrolase, AtNUDX19, impacts on the pyridine nucleotides status and confers photooxidative stress tolerance. *Sci. Rep*. **6**, 37432; doi: 10.1038/srep37432 (2016).

**Publisher’s note**: Springer Nature remains neutral with regard to jurisdictional claims in published maps and institutional affiliations.

## Supplementary Material

Supplementary Information

## Figures and Tables

**Table 1:** Levels and redox states of pyridine nucleotides in *nudx19* mutants.

	Normal light			High light (6 h)		
	Wild type	KD-*nudx19*	KO-*nudx19*	wild type	KD-*nudx19*	KO-*nudx19*
NADPH	1.24	2.00	2.15	1.19	2.25	2.75
NADP^+^	6.86	4.11	4.05	7.09	6.33	6.47
NADH	3.55	3.24	3.27	5.86	5.65	5.14
NAD^+^	6.94	6.31	6.19	8.74	5.82	6.27
NADP(H)	8.10	6.11	6.2	8.28	8.58	9.22
NAD(H)	10.49	9.55	9.46	14.60	11.47	11.41
NAD(P)(H)	18.59	15.66	15.66	22.88	20.05	20.63
NADPH/NADP(H)	0.15	0.33	0.35	0.14	0.26	0.30
NADH/NAD(H)	0.34	0.34	0.35	0.40	0.49	0.45
NADP(H)/NAD(H)	0.77	0.64	0.66	0.57	0.75	0.81

Total levels of NAD(H) and NADP(H) (nmol g^−1^ FW) and their redox states were calculated from the data shown in Fig. 2.
