# Supplementary material for: Loss-of-function of an Arabidopsis NADPH pyrophosphohydrolase, AtNUDX19, impacts on the pyridine nucleotides status and confers photooxidative stress tolerance
Source: Sci Rep. 2016 Nov 22;6:37432. doi: 10.1038/srep37432 (PMC5118724; doi:10.1038/srep37432)
Supplement: Supplementary Information [file srep37432-s1.pdf]

Title: Loss-of-function of an *Arabidopsis* NADPH pyrophosphohydrolase, AtNUDX19, impacts on the pyridine nucleotides status and confers photooxidative stress tolerance

Authors: Takanori Maruta[1,2], Takahisa Ogawa[1,2], Masaki Tsujimura[1], Keisuke Ikemoto[1], Tomofumi Yoshida[1], Hiro Takahashi[3], Kazuya Yoshimura[4], and Shigeru Shigeoka[1,*]

Affiliations:

[1]Department of Advanced Bioscience, Faculty of Agriculture, Kindai University, 3327-204 Nakamachi, Nara 631-8505, Japan

[2]Department of Life Science and Biotechnology, Faculty of Life and Environmental Science, Shimane University, 1060 Nishikawatsu, Matsue, Shimane 690-8504, Japan

[3]Graduate School of Horticulture, Chiba University, 648 Matsudo, Matsudo, Chiba 271-8510, Japan

[4]Department of Food and Nutritional Science, College of Bioscience and Biotechnology, Chubu University, 1200 Matsumoto-cho, Kasugai, Aichi 487-8501, Japan (K.Y.)

[*]Corresponding author: Prof. Shigeru Shigeoka; Tel/Fax, +81-742-43-8083; E-mail, shigeoka@nara.kindai.ac.jp

Supplementary Figures: S1 to S4
Supplementary Tables: S1 and S2

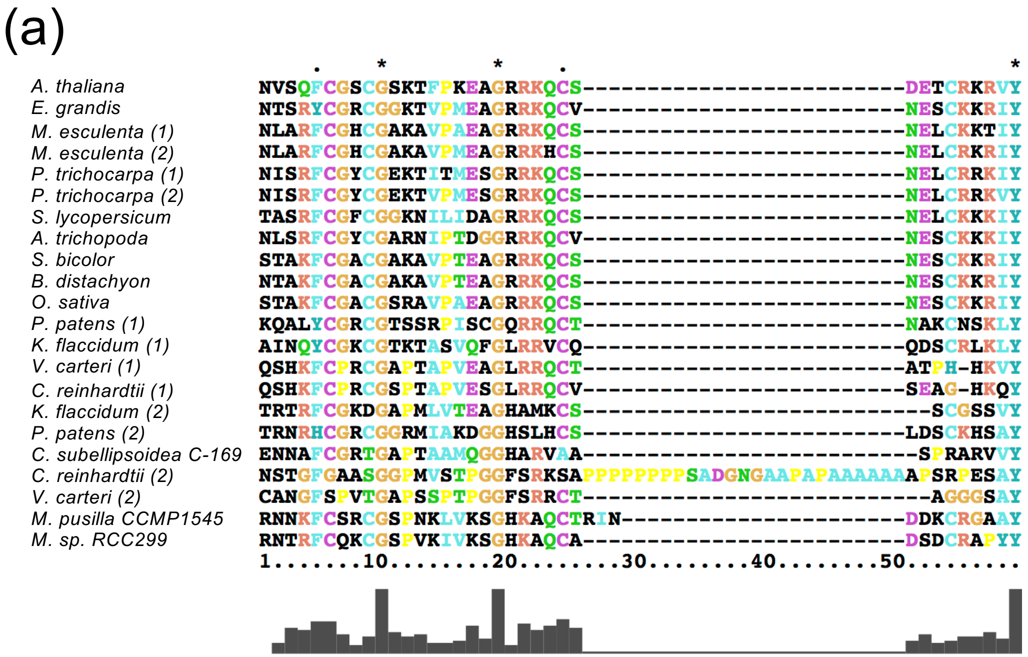

Figure S1 Alignment of plant SQPWP enzymes.

Alignments of zf-NADH-PPase (a) and partial NUDIX (b) domains of plant SQPWP enzymes were made using ClustalX software.

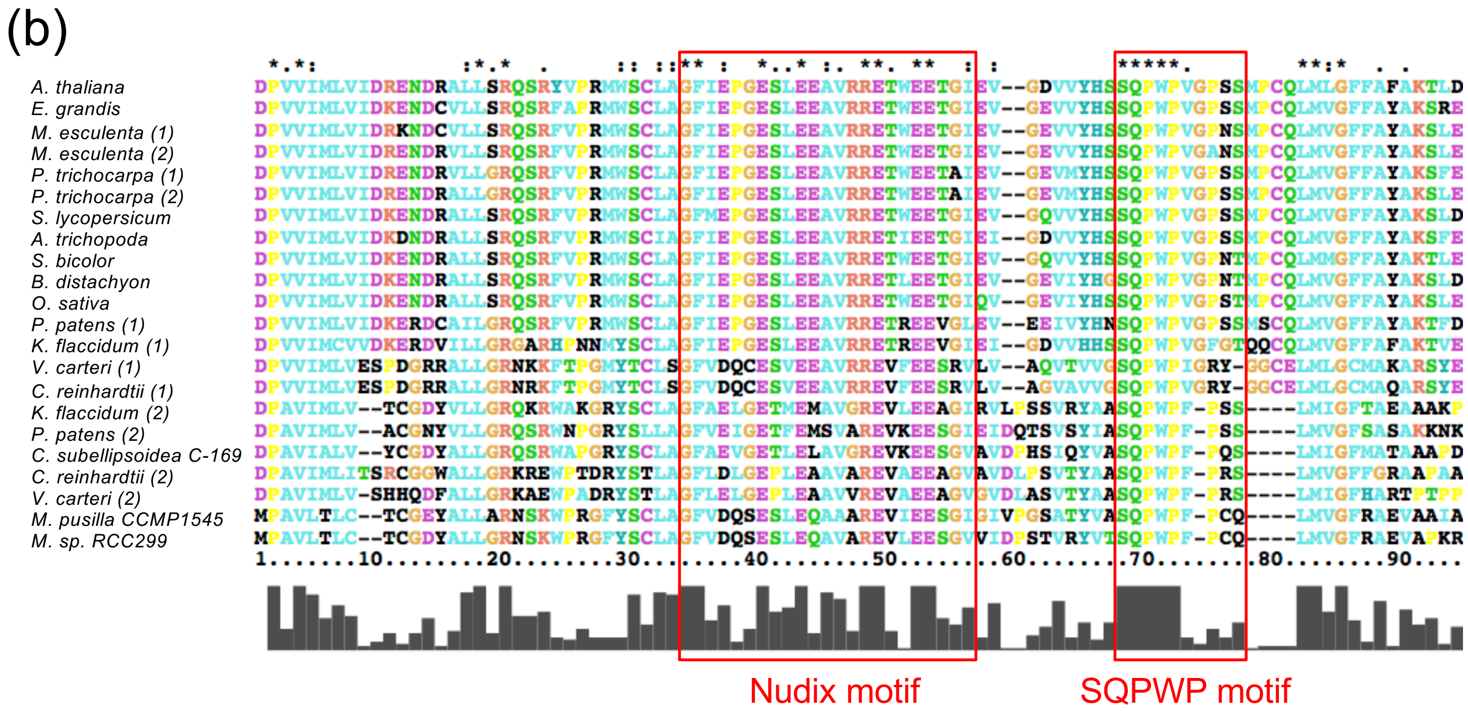

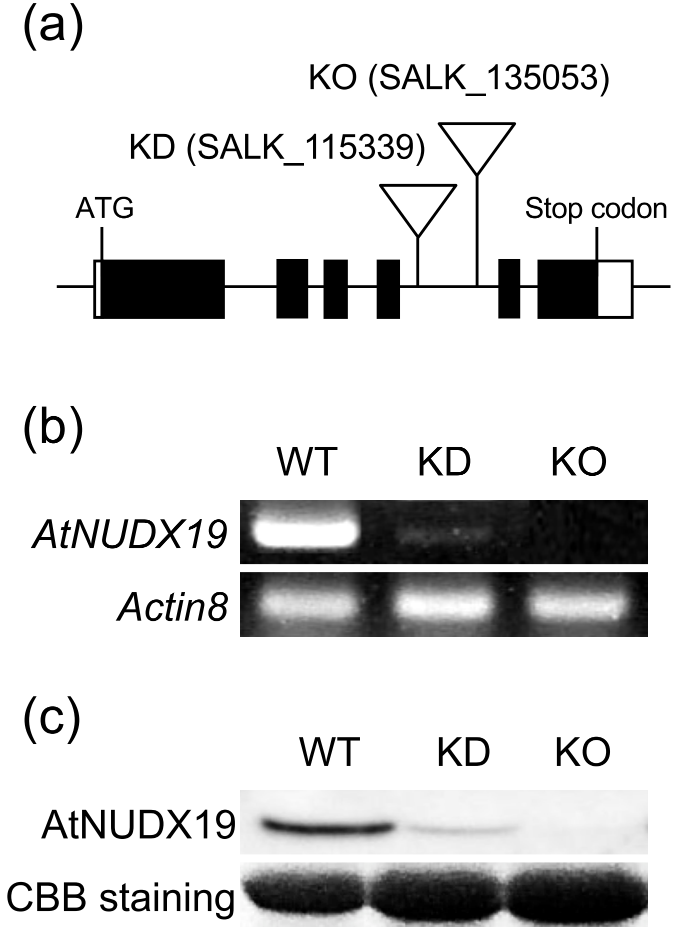

Figure S2 Knockout and knockdown mutants of AtNUDX19.
(a) Molecular structure and T-DNA insertion sites of the *AtNUDX19* gene (SALK_135053 and SALK_115339). T-DNA insertion sites are indicated with triangles, and black and white boxes represent exons and untranslational regions, respectively. (b) Semi-quantitative RT-PCR and (c) western blotting data showing the expression levels of AtNUDX19 in the leaves of 3-week-old wild-type and mutant plants under normal growth conditions.

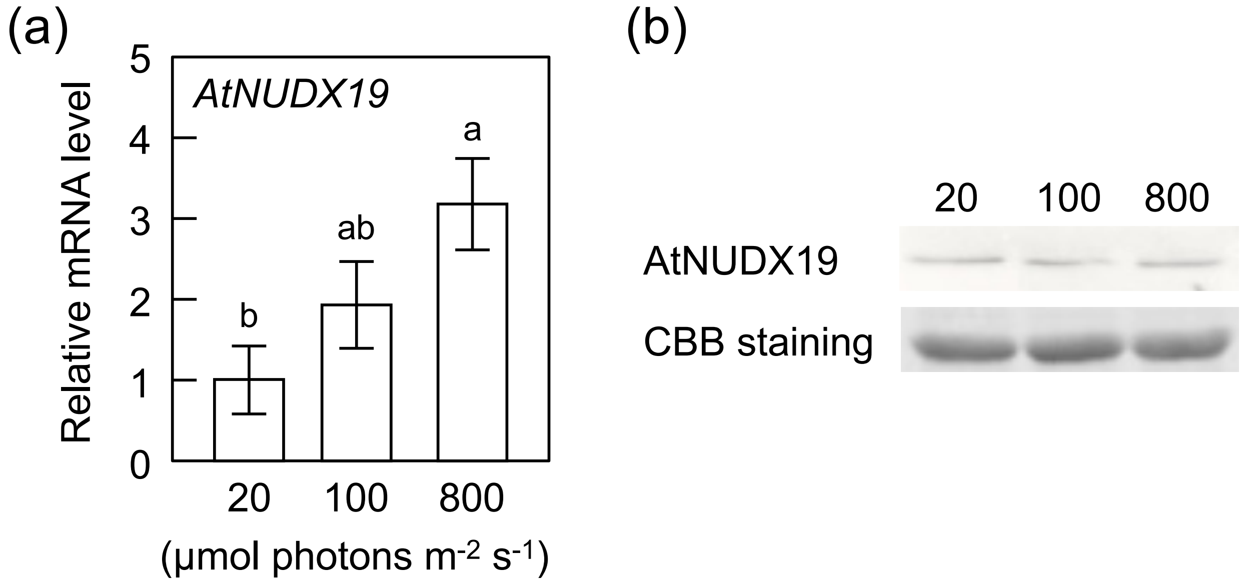

Figure S3 Effects of growth light intensity on the AtNUDX19 expression. One-week-old wild-type plants were further grown under different light intensities (16 h of 20, 100, or 800 μmol photons m^{-2} s^{-1}, 8 h of dark) for 2 weeks. Transcript (a) and protein (b) levels of AtNUDX19 in leaves were investigated by q-PCR and western blotting, respectively. Data are means ± SD for at least 3 individual experiments (≥5 plants for 1 experiment). Values without a common letter were significantly different according to the t-test ($P < 0.05$).

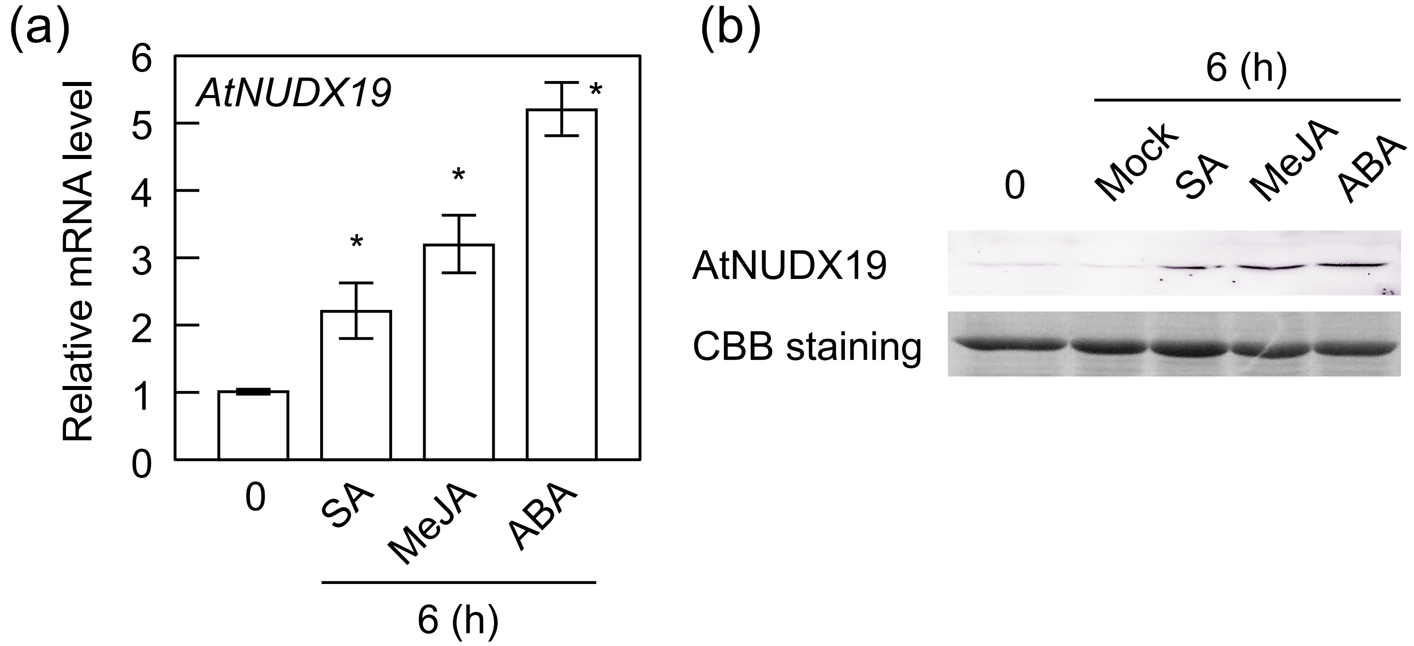

Figure S4 Effect of hormones on the AtNUDX19 expression.
Three-week-old wild-type and mutant plants were treated with water, SA (100 μM), MeJA (25 μM), and ABA (35 μM) for 6 h. Transcript (a) and protein (b) levels of AtNUDX19 in leaves were investigated by q-PCR and western blotting, respectively. Data are means ± SD for at least 3 individual experiments (>5 plants for 1 experiment). Significant differences: *$P < 0.05$ vs. the value at 0 h.

Supplemental Table S1

Sub-cellular localization of SPQWP enzymes was predicted using TargetP 1.1 (Emanuelsson et al., 2000) and WoLF PSORT (Horton et al., 2007). 'C-ter. 3 AA' shows last 3 amino acids sequence, and potential PTS sequences are in red. When domain structure was analyzed using Pfam, Potri.018G070400.1 and Pp3c3_2090V3.1 lacked the zf-NADH-PPase domain. However, sequence alignment obviously showed an existence of the domain in these proteins (see Supplemental Figure S1).

Group	Organism	Accession number (or gene name)	AA size	Predected localization		C-ter. 3 AA	Position of Pfam domains (AA)				
				Target P	WoLF PSORT		Ank_2	NUDIX-like	zf-NADH-PPase	NUDIX	Oncus
	Arabidopsis thaliana	AtNUDX19	438	Chl: 0.470	Chl: 8.5, Chl_Mit: 7.5, Mit: 5.5	SSL	-	87-205	209-240	244-369	-
	Eucalyptus grandis	Eucgr.C04181.1	517	Other: 0.706	Nuc:13	SNL	-	163-282	284-317	319-446	-
	Manihot esculenta (1)	Manes.06G050000.1	444	Chl: 0.619	Chl: 14	SNL	-	91-209	211-243	246-373	-
	Manihot esculenta (2)	Manes.14G130500.1	444	Chl: 0.637	Chl: 12.5, Chl_Mit: 7.5	SNL	-	90-209	211-244	246-373	-
	Populus trichocarpa (1)	Potri.018G070400.1	450	Chl: 0.491	Chl: 10	SNL	-	96-215	(219-250)	253-380	-
	Populus trichocarpa (2)	Potri.006G154200.1	407	Chl: 0.856	Nuc: 6, Chl: 5	SNL	-	51-172	176-207	210-337	-
	Solanum lycopersicum	Solyc06g075080.2.1	458	Mit: 0.565	Chl: 11.5, Chl_Mit: 7.5	TNN	-	83-204	207-239	241-367	-
Subgroup A	*Amborella trichopoda*	evm_27.model.AmTr_v1.0_scaffold00081.27	447	Other: 0.224	Chl: 12	SNL	-	92-216	218-251	254-380	-
	Sorghum bicolor	Sobic.010G030700.1	402	Mit: 0.415	Chl: 6, Mit: 5	SNL	-	52-175	177-210	213-338	-
	Brachypodium distachyon	Bradi1g51060.1	394	Mit: 0.253	Chl: 5	SNL	-	44-167	169-202	204-330	-
	Oryza sativa	LOC_Os06g04910.1	405	Mit: 0.363	Chl: 6, Mit: 6	SNL	-	56-178	180-213	215-342	-
	Physcomitrella patens (1)	Pp3c3_2090V3.1	388	Other: 0.846	Chl: 5, Nuc: 5	SNY	-	39-158	(162-193)	195-321	-
	Klebsormidium flaccidum (1)	kfl00050_0140	403	Other: 0.456	Per: 6	SRV	-	36-179	183-214	216-342	-
	Volvox carteri (1)	Vocar.0008s0194.1	575	Chl: 0.750	Nuc: 4.5	GRL	-	128-240	242-271	276-400	-
	Chlamydomonas reinhardtii (1)	Cre09.g396900.t1.2	463	Chl: 0.479	Chl: 5	AKL	-	83-210	212-244	247-373	-
	Klebsormidium flaccidum (2)	kfl00032_0450	541	Mit: 0.609	Chl: 14	EGY	-	67-177	179-210	212-315	429-532
	Physcomitrella patens (2)	Pp3c14_12120V3.1	533	Mit: 0.657	Chl: 14	AWG	-	78-204	206-239	241-386	424-527
	Coccomyxa subellipsoidea C-169	estExt_fgenesh1_pg.C_220093	551	Mit: 0.951	Chl: 11	SGY	-	78-181	-	217-372	437-543
Subgroup B	*Chlamydomonas reinhardtii* (2)	Cre13.g582476.t1.1	750	Mit: 0.879	Mit: 8, Chl: 6	EGY	-	106-244	-	306-409	629-739
	Volvox carteri (2)	Vocar.0001s0115.1	627	Mit: 0.878	Mit: 8.5, Chl_Mit: 7.5, Chl: 5.5	DGY	-	124-243	-	277-399	508-616
	Micromonas pusilla CCMP1545	MicpuC2.EuGene.0000120481	534	Chl: 0.914	Chl: 11.5, Chl_Mit: 7.3	ADP	-	-	275-307	313-411	-
	Micromonas sp. RCC299	EuGene.1400010022	642	Chl: 0.883	Chl: 11	YQY	-	110-247	249-282	284-435	-

Supplemental Table S1 Transcriptome data of the wild-type and KO-*nudx19* plants.

Up-regulated genes in KO-*nudx19*

Probe ID	AGI code	Gene	Annotation	Experiment 1			Experiment 2			Average
				KO/WT (log2)	*p* value	*q* value	KO/WT (log2)	*p* value	*q* value	KO/WT (log2)
258427_at	AT3G16600		SNF2 domain-containing protein / helicase domain-containing protein	5.1	7.8E-03	1.7E-01	2.0	6.5E-03	1.5E-01	3.6
265732_at	AT2G01300		Unknown protein	3.9	7.7E-04	3.2E-02	3.1	5.9E-03	1.4E-01	3.5
258779_at	AT3G11870		Endoribonuclease/protein kinase IRE1-like	2.5	9.7E-04	3.7E-02	0.6	7.8E-03	1.7E-01	1.6
251625_at	AT3G57260	PR2	Pathogenesis-related gene 2	1.1	2.3E-05	3.3E-03	2.0	2.0E-05	4.0E-03	1.6
266385_at	AT2G14610	PR1	Pathogenesis-related gene 1	1.4	3.0E-05	3.6E-03	1.4	2.3E-05	4.0E-03	1.4
258947_at	AT3G01830		Calcium-binding EF-hand family protein	1.4	4.5E-03	1.1E-01	1.4	1.3E-04	1.0E-02	1.4
245755_at	AT1G35210		Unknown protein	1.4	9.3E-03	1.9E-01	1.2	2.8E-03	8.3E-02	1.3
252136_at	AT3G50770	CML41	Calmodulin-like 41	1.3	1.3E-03	4.7E-02	1.2	2.1E-04	1.4E-02	1.3
264958_at	AT1G76960		Unknown protein	0.9	2.5E-03	7.5E-02	1.4	2.3E-05	4.0E-03	1.2
265067_at	AT1G03850	GRXS13	Glutaredoxin family protein	1.4	2.3E-05	3.3E-03	0.7	3.7E-03	1.0E-01	1.1
248676_at	AT5G48850	ATSDI1	Tetratricopeptide repeat (TPR)-like superfamily protein	0.8	3.1E-04	1.6E-02	1.3	5.4E-03	1.3E-01	1.1
246098_at	AT5G20400		2-oxoglutarate and Fe(II)-dependent oxygenase superfamily protein	0.9	7.8E-05	7.0E-03	1.1	3.7E-03	1.0E-01	1.0
267209_at	AT2G30930		Unknown protein	0.7	2.0E-05	3.3E-03	1.3	2.0E-05	4.0E-03	1.0
249890_at	AT5G22570	WRKY38	WRKY DNA-binding protein 38	0.9	1.1E-03	4.0E-02	1.0	2.1E-04	1.4E-02	1.0
260046_at	AT1G73805	SARD1	SAR Deficient 1	0.8	1.8E-03	5.9E-02	1.1	3.4E-03	9.6E-02	1.0
247327_at	AT5G64120	PRX71	Peroxidase 71	0.8	2.0E-05	3.3E-03	1.1	2.0E-05	4.0E-03	1.0
264680_at	AT1G65510		Unknown protein	1.0	5.5E-04	2.5E-02	0.8	1.7E-03	5.7E-02	0.9
245252_at	AT4G17500	ERF-1	Ethylene responsive element binding factor 1	0.9	7.8E-05	7.0E-03	0.9	2.0E-05	4.0E-03	0.9
247925_at	AT5G57560	XTH22	Xyloglucan endotransglucosylase 22	0.7	2.0E-05	3.3E-03	1.1	2.0E-05	4.0E-03	0.9
247013_at	AT5G67480	BT4	BTB and TAZ domain protein 4	0.7	4.0E-05	4.4E-03	1.0	2.0E-05	4.0E-03	0.9
262958_at	AT1G54410		Dehydrin family protein	0.6	2.0E-05	3.3E-03	1.1	2.0E-05	4.0E-03	0.9
262118_at	AT1G02850	BGLU11	Beta glucosidase 11	0.9	3.5E-04	1.8E-02	0.7	8.8E-05	7.8E-03	0.8
263584_at	AT2G17040	NAC036	NAC domain containing protein 36	0.9	5.5E-04	2.5E-02	0.7	8.7E-04	3.7E-02	0.8
256964_at	AT3G13520	AGP12	Arabinogalactan protein 12	0.8	4.0E-05	4.4E-03	0.8	6.0E-05	6.2E-03	0.8
264635_at	AT1G65500		Unknown protein	0.7	7.8E-03	1.7E-01	0.9	7.7E-04	3.4E-02	0.8
263539_at	AT2G24850	TAT3	Tyrosine aminotransferase 3	0.6	9.3E-03	1.9E-01	1.0	2.0E-05	4.0E-03	0.8
248186_at	AT5G53880		Unknown protein	0.6	2.0E-05	3.3E-03	1.0	2.0E-05	4.0E-03	0.8
262314_at	AT1G70810		Calcium-dependent lipid-binding family protein	0.8	2.0E-05	3.3E-03	0.7	1.1E-04	9.5E-03	0.8
259664_at	AT1G55330	AGP21	Arabinogalactan protein 21	0.7	3.0E-05	3.6E-03	0.7	2.3E-05	4.0E-03	0.7
265615_at	AT2G25450		2-oxoglutarate and Fe(II)-dependent oxygenase superfamily protein	0.7	2.0E-05	3.3E-03	0.6	8.8E-05	7.8E-03	0.7
260541_at	AT2G43530		Scorpion toxin-like knottin superfamily protein	0.7	4.9E-04	2.3E-02	0.6	7.7E-04	3.4E-02	0.7
250722_at	AT5G06190		Unknown protein	0.7	3.7E-03	9.9E-02	0.6	4.9E-04	2.5E-02	0.7
252563_at	AT3G45970	ATEXLA1	Expansin-like A1	0.6	2.7E-04	1.5E-02	0.7	6.9E-04	3.2E-02	0.7
249850_at	AT5G23240	DJC76	DNAJ heat shock N-terminal domain-containing protein	0.6	1.1E-03	4.0E-02	0.7	1.9E-04	1.3E-02	0.7
249754_at	AT5G24530	DMR6	Downy Mildew Resistant 6	0.6	6.9E-04	2.9E-02	0.7	2.0E-05	4.0E-03	0.7
257054_at	AT3G15353	MT3	Metallothionein 3	0.6	2.0E-05	3.3E-03	0.6	4.4E-04	2.3E-02	0.6
249527_at	AT5G38710		Methylenetetrahydrofolate reductase family protein	0.6	1.5E-04	1.0E-02	0.6	6.2E-04	3.0E-02	0.6

Down-regulated genes in KO-*nudx19*

Probe ID	AGI code	Gene	Annotation	Experiment 1			Experiment 2			Average
				KO/WT (log2)	*p* value	*q* value	KO/WT (log2)	*p* value	*q* value	KO/WT (log2)
260869_at	AT1G43800		Plant stearoyl-acyl-carrier-protein desaturase family protein	-2.9	2.2E-03	7.1E-02	-2.8	1.2E-03	6.0E-02	-2.9
266415_at	AT2G38530	LTP2	Lipid transfer protein 2	-1.4	2.3E-05	6.0E-03	-0.8	4.6E-05	1.0E-02	-1.1
263161_at	AT1G54020		GDSL-like Lipase/Acylhydrolase superfamily protein	-1.0	1.1E-04	1.3E-02	-1.1	2.0E-05	8.7E-03	-1.1
247573_at	AT5G61160	AACT1	Anthocyanin 5-aromatic acyltransferase 1	-0.6	9.7E-04	4.2E-02	-1.4	4.6E-05	1.0E-02	-1.0
254819_at	AT4G12500		Bifunctional inhibitor/lipid-transfer protein/seed storage 2S albumin superfamily protein	-0.8	4.9E-03	1.1E-01	-1.2	2.0E-05	8.7E-03	-1.0
266142_at	AT2G39030	NATA1	Acyl-CoA N-acyltransferases (NAT) superfamily protein	-1.0	7.7E-04	3.8E-02	-1.0	5.2E-05	1.1E-02	-1.0
252607_at	AT3G44990	ATXTR8	XYLOGLUCAN ENDO-TRANSGLYCOSYLASE-RELATED 8	-1.4	2.0E-05	6.0E-03	-0.6	2.0E-05	8.7E-03	-1.0
266989_at	AT2G39330	JAL23	Jacalin-related lectin 23	-1.1	5.5E-04	3.1E-02	-0.6	1.5E-04	2.0E-02	-0.9
263549_at	AT2G21650	RSM1	Homeodomain-like superfamily protein	-0.6	3.7E-03	9.4E-02	-1.0	7.8E-05	1.3E-02	-0.8
249732_at	AT5G24420	PGL5	6-phosphogluconolactonase 5	-0.6	2.3E-05	6.0E-03	-1.0	2.0E-05	8.7E-03	-0.8
265892_at	AT2G15020		Unknown protein	-0.6	2.5E-03	7.5E-02	-0.9	7.7E-04	4.8E-02	-0.8
248812_at	AT5G47330		Alpha/beta-Hydrolases superfamily protein	-0.9	1.2E-02	2.0E-01	-0.6	8.5E-03	1.9E-01	-0.8
260523_at	AT2G41720	EMB2654	Tetratricopeptide repeat (TPR)-like superfamily protein	-0.6	1.8E-03	6.2E-02	-0.7	7.1E-03	1.7E-01	-0.7
258034_at	AT3G21300		RNA methyltransferase family protein	-0.6	3.4E-03	8.9E-02	-0.7	6.8E-05	1.3E-02	-0.7
250880_at	AT5G04070		NAD(P)-binding Rossmann-fold superfamily protein	-0.6	7.1E-03	1.4E-01	-0.7	1.7E-03	7.1E-02	-0.7
251119_at	AT3G63510		FMN-linked oxidoreductases superfamily protein	-0.7	4.6E-05	7.9E-03	-0.6	1.1E-03	5.8E-02	-0.7
